# The effect of hypocaloric diet enriched in legumes with or without L-arginine and selenium on anthropometric measures in central obese women

**Published:** 2010

**Authors:** Mohammad Alizadeh, Sevana Daneghian, Aida Ghaffari, Alireza Ostadrahimi, Abdolrasoul Safaeiyan, Rassul Estakhri, Bahram Pourghasem Gargari

**Affiliations:** a Student Research Committee, Tabriz University of Medical Sciences, Tabriz, Iran; b Nutrition Research Center, Department of Nutrition, Faculty of Health and Nutrition, Tabriz University of Medical Sciences, Tabriz, Iran; c Department of Vital Statistics and Epidemiology, Faculty of Health and Nutrition, Tabriz University of Medical Sciences, Tabriz, Iran; d Liver and Gastrointestinal Disease Research Center, Tabriz University of Medical Sciences, Tabriz, Iran

**Keywords:** Arginine, Selenium, Diet, Reducting, Fabaceae, Obesity, Abdominal

## Abstract

**BACKGROUND:**

Identifying new ways to decrease adiposity will be very valuable for health. The aim of this study was to find out whether L-Arginine (Arg) and selenium alone or together can increase the effect of hypocaloric diet enriched in legumes (HDEL) on anthropometric measures in healthy obese women.

**METHODS:**

This randomized, double-blind, placebo-controlled trial was undertaken in 84 healthy premenopausal women with central obesity. After 2 weeks of run-in on an isocaloric diet, participants were randomly considered to eat HDEL, Arg (5 g/d) and HDEL, selenium (200 µg/d) and HDEL or Arg, selenium and HDEL for 6 weeks. The following variables were assessed before intervention and 3 and 6 weeks after it: weight, waist circumference, hip circumference, waist to hip ratio (WHR), body mass index (BMI), and fasting nitrite/nitrate (NO_x_) concentrations. Other variables (arm, thigh, calf and breast circumferences, subscapular, triceps, biceps and suprailiac skinfold thicknesses, sum of skinfold thicknesses (SSF), body density (D) and estimated percent of body fat (EPF)) were assessed before and after intervention.

**RESULTS:**

HDEL showed a significant effect in reduction of waist, hip, arm, thigh, calf and breast circumferences, triceps, biceps, subscapular and suprailiac skinfold thicknesses, WHR, SSF, D and EPF. HDEL + Arg + selenium significantly reduced suprailiac skinfold thicknesses; and there was no significant effect of HDEL, Arg, selenium and Arg plus selenium on weight, BMI and fasting NO_x_.

**CONCLUSIONS:**

The study indicates that HDEL + Arg + selenium reduce suprailiac skinfold thicknesses which represents the abdominal obesity reduction.

Obesity is increasingly prevailing worldwide.^1^ In the world, more than one billion adults are overweight and over 300 million are obese.^2^ Obesity causes main health difficulties such as heart disease, diabetes mellitus, cancer, stroke, impacts on physical and mental health, quality of life, and cost of life.^3^ In Iran the prevalence of overweight among urban and rural 15-39 years old people is estimated about 22% and 16%, respectively. Equivalent percents in 40-69 years old people are 40% and 26%.^4^ On the other hand there is a particular type of obesity in Iran, the so-called Middle-Eastern pattern which increases the risk of obesity-related co-morbidities.^5^ It is necessary to treat obesity both with lifestyle modifications and/or pharmacological therapy.^6^ Unfortunately, there are few ways to manage the epidemic obesity, because the existing anti-obesity drugs are not significantly effective and have many side effects.^7^ Since even modest weight loss (5-10%) in obese subjects can highly improve the risk of cardiovascular disease and diabetes, identifying new ways to decrease body fat will be very valuable for health.^7^

The administration of L-Arginine, a nitric oxide (NO) precursor, reduced adipose tissue in zucker diabetic fatty (ZDF) rats,^8^ dietinduced obese rats,^9^ growing-finishing pigs^10^ and type 2 diabetic humans.^11^ The effect of low dose L-Arginine in healthy obese women was not clear. Some evidence showed that NO affected metabolism of adipose tissue.^12^ Physiological concentrations of NO enhanced glucose and fatty acid oxidation and inhibited glucose and triglyceride synthesis.^13^ It was not clear whether low dose of oral L-Arginine supplementation can increase fasting serum nitrite/nitrate (NO_x_) concentrations or not. As a high reactive oxidant, high concentrations of NO formed by inducible nitric oxide synthase (iNOS) can disrupt cell action.^14^ Obesity increased iNOS expression and overproduced NO.^15^,^16^ Although serum concentrations of NO were higher in the overweight and obese people,^17^ due to an increased free radical production,^18^ its availability was low.^19^,^20^ Serum physiologic concentration of NO in normal adults is in the range of 25-35 µmol/l.^17^ This is produced by eNOS and nNOS.^21^ Serum concentration of NO in normal weight persons is near the minimum end of the range and in overweight persons near the maximum end of it.^17^ When an immunological stimulus such as bacterial endotoxin or inflammatory cytokines, stimulate macrophages, iNOS is highly expressed in many types of cells and it produces a large amount of NO and serum concentrations of NO reach a pathologic amount.^21^ For example, the production of NO by unstimulated bovine venular endothelial cells is only approximately 7% of that by lipopolysaccharide-activated raw 264.7 macrophages.^13^ Similarly, in vivo systemic production of NO increases 15-fold in response to immunological challenge in rats.^22^

It seems that selenium, as an antioxidant, can increase the availability of NO. Selenium decreased lipopolysaccharide-induced NO production (pathologic amount) in murine macrophage cultures in vitro^23^ and the deficiency of selenium enhanced iNOS expression in RAW 264.7 macrophages. Also there was a contrary association between cellular selenium concentrations and the expression of iNOS in RAW 264.7 cells stimulated by lipopolysaccharide.^24^

In this study a basic diet for all participants was needed. Legume is one of the healthy dietary pattern components.^5^ High legumes consumption may decrease the risk of general and central obesity.^5^,^25^ Recently, a study showed higher weight loss in those volunteers who followed the legumes enriched hypocaloric diet compared to hypocaloric diet without legumes.^26^ On the other hand legumes are cheap and rich sources of L-Arginine and selenium. With HDEL and L-Arginine supplementation, total intake of L-Arginine could be increased about 8.3 gr/day; the minimum amount in previous studies, which had an effect on body fat loss.

Regarding poor compliance of high doses of L-Arginine, this is the first time that the effect of low dose of L-Arginine, selenium, and HDEL, alone or together, on anthropometric measures and fasting NO_x_ concentrations in central obese women is studied. The purpose of the study was to find out whether L-Arginine and selenium, alone or together, can increase the effect of HDEL. L-Arginine was administered to enhance physiologic level of NO, and selenium was administered to ameliorate antioxidant defense, to increase NO availability and to prevent pathologic production of NO, while HDEL was administered as a rich source of L-Arginine and selenium and as the base of the treatment for obesity.

## Methods

### 

#### Participants

**Figure 1:**
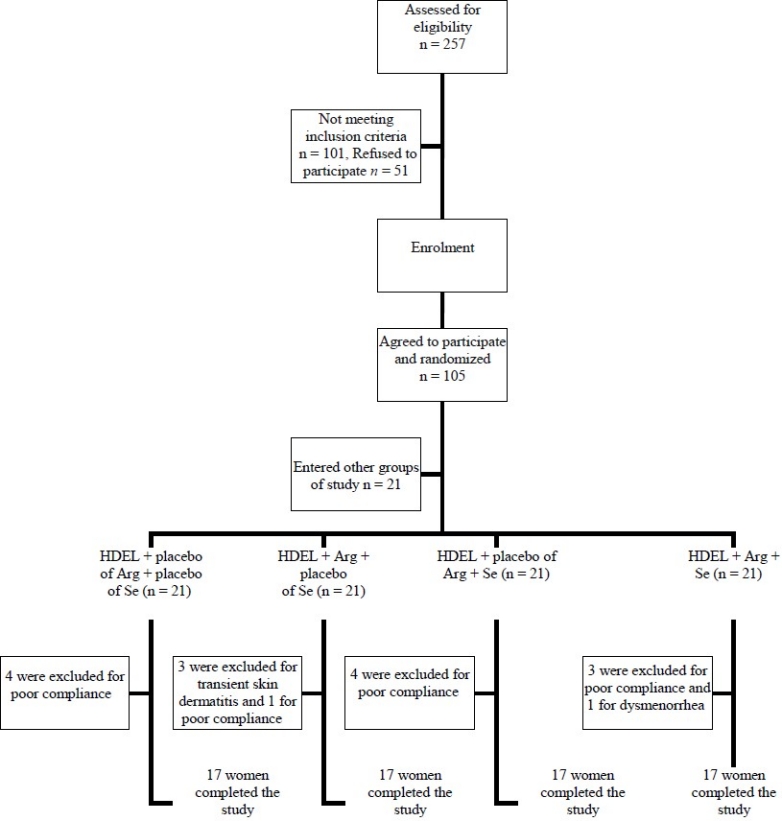
Study flow diagram of participants. HDEL: Hypocaloric Diet Enriched in Legumes; Arg: L-Arginine; Se: Selenium

The study was approved by the ethics committee of Tabriz University of Medical Sciences, and registered at www.irct.ir (irct ID: irct138712101720N1). A written informed consent was taken from all the selected subjects. It was a randomized, double-blind, placebo-controlled study which lasted for 8 weeks. Only female subjects were selected because of interactions between NO and estrogens. Study candidates were called in using local and newspaper advertisements. A total of 257 premenopausal women were screened to enter the study. Inclusion criteria were age factor (premenopausal women aged from 20 to 50 years), waist circumference (women with waist circumference (WC) higher than 88 cm), no participation in weight-reduction programs, and maintenance of a stable weight (± 2 kg) during the last 6 months. The following are the exclusion criteria: any secondary cause of hyperglycemia (as trauma) or hypertension (as renal disease); treatment with oral hypoglycemic agents or insulin, antilipemic or antihypertensive drugs; taking any vitamin or mineral supplements, nitrate, L-Arginine, selenium or antacids containing magnesium or calcium; having psychiatric disorders; having untreated hypothyroidism; having cancer or hepatic, systemic, pulmonary, renal or cardiovascular disease; presence of inflammatory or infectious disease; smoking; alcoholism and legumes intolerance. Finally, a total of 84 premenopausal women were enrolled but sixteen of them were excluded and did not complete the study: three of them because of transient skin dermatitis in L-Arginine group and one because of dysmenorrhea in L-Arginine plus selenium group and the rest for the sake of poor compliance. Sixty eight women remained for the present analysis. All the participants were selected from the outpatient clinic of Tabriz University of Medical Sciences, Iran. Figure 1 shows the flow chart of the participants of the study.

#### Diets

The caloric needs for each subject separately was determined by the equation from the Institute of Medicine, Food, and Nutrition board.^27^ In the run-in period the participants consumed isocaloric diet for 2 weeks (Table 1). In the in tervention period the members of all of the groups ate HDEL, which had 2 servings/day legumes instead of meat (Table 2). The composition of all diets was 55% carbohydrate, 30% fat and 15% protein. All participants in all groups were given a diet of 500 kcal less than their caloric needs in intervention period. The participants were being visited weekly; each session for every participant lasted for 20-30 minutes. Behavioral counseling was made. The nutritionist described the advantages of diets for the participants and told them that the central obesity might be controlled by continuing the diets. The diets were prescribed individually by a calorie count system. Also an exchange list was educated to each participant for calorie counting and exchanging food items. A nutritionist taught participants how to write food diaries. Each participant had to write her 3-day diet and physical activity records before run in, and before, in the middle and at the end of the intervention. The dairies were evaluated by the investigators. A 7-day menu with 42 meals and snacks at seventeen calorie levels (1200 to 2800) was developed for each diet. The participants’ compliance was evaluated by reviewing the 3-day food records and the weekly visits. Every week the number of reported food group exchanges, was compared with prescribed exchanges. Participants who ran at least 80% of the planned diets were encouraged to follow it seriously for the next week. Volunteers who did not run at least 80% of prescribed diets for two successive weeks, were excluded from the study (n = 12). L-Arginine and selenium supplements were delivered weekly and participants were encouraged to use them regularly. Unused tablets were counted at each visit and were collected at the end of the treatment period to measure the compliance. Subjects consumed 96 ± 3.5% of the pills assigned.

**Table 1:** Isocaloric diets in run in period

kcal	Milk (ex)	Vegetable (ex)	Fruit (ex)	Meat (ex)	Cereal (ex)	Sugar (ex)	Fat (ex)
1800	2	3	3	3	9	2	6
1900	2	3	3	3	10	2	7
2000	2	3	4	3	10	2	8
2100	2	3	4	3	11	2	8.5
2200	2	3	4	4	11	2.5	8
2300	2	3	4	4	12	2.5	9
2400	2	3	4	4	13	2.5	10
2500	2	3	5	4.5	13	2.5	10

Ex: The Exchange Lists are the basis of a meal planning system designed by the committee of the American Diabetes Association and the American Dietetic Association. American Diabetes Association, Inc., the American Dietetic Association

**Table 2:** Hypocaloric diets enriched in legumes in intervention period

kcal	Milk (ex)	Vegetable (ex)	Fruit (ex)	Meat without legume (ex)	Cereal (ex) + legume (ex)	Sugar (ex)	Fat (ex)
1200	2	2	3	-	2 + 2	2	6
1300	2	2	3	-	3 + 2	1.5	6
1400	2	2	3	-	4 + 2	1.5	7
1500	2	3	3	0.5	4 + 2	2	7
1600	2	3	3	1	5 + 2	2	7
1700	2	3	3	1	6 + 2	2	8
1800	2	3	3	1	7 + 2	2	8
1900	2	3	3	1	8 + 2	2	9
2000	2	3	4	1	8 + 2	2	10

Ex: The Exchange Lists are the basis of a meal planning system designed by the committee of the American Diabetes Association and the American Dietetic Association. American Diabetes Association, Inc., the American Dietetic Association.

#### Study Procedures

During the 8 weeks of the study, the clinic had contact with the subjects weekly. The true isocaloric needs of some of the persons are different from the determined amount in the equation from the Institute of Medicine, Food, and Nutrition Board. In such individuals, isocaloric diet will cause weight reduction or weight gain rather than weight maintenance. Such people could cause errors in the study. So among the individuals who were eligible to enter the study, just those were selected who kept their weight at the end of the run-in period with isocaloric diet calculated according to the equation. Moreover, the dietary pattern of each participant was different and could affect the results of interventions. Hence, in addition to getting detailed information about the subjects, a run-in period was planned to make similarity in the macronutrients consumption. After 2 weeks of run-in period on an isocaloric diet, subjects were randomly assigned to four groups: 1) HDEL supplemented with placebo of L-Arginine and placebo of selenium, 2) HDEL supplemented with L-Arginine (5 gr/day) and placebo of selenium, 3) HDEL supplemented with selenium (200 µg/day) and placebo of L-Arginine and 4) HDEL supplemented with L-Arginine and selenium in intervention period for 6 weeks. For allocation of the participants, a computer-generated list of random numbers was used. For homogenizing 4 groups according to the general characteristics, all anthropometric measures and NO_x_ concentrations, participants were randomly allocated to 4 groups several times and selected the most homogenous groups. The measurements were obtained before, in the middle and at the end of the intervention.

L-Arginine (5 gr/d) was administered in the form of 1 gr L-Arginine HCL tablet (Pooyan nutrition Co, Iran, joint venture with Mass Global Nutrition, Canada); 2 tablet/TID with meals. Selenium (200 µg/d) was administered in the form of selenium enriched yeast tablet (Nature Made Pharmavite LLC, USA); one tablet/day, 2 hours after one of the meals (and L-Arginine). The composition of the placebo tablets was 69.5% starch, 30% lactose, and 0.5% magnesium stearate. The participants were told not to vary their usual physical activity during the study.

#### Measurements

The following variables were assessed before the intervention (after run-in) and 3 and 6 weeks after it: weight, waist circumference, hip circumference, waist to hip ratio (WHR), body mass index (BMI), and fasting nitrite/nitrate (NO_x_) concentrations. Other variables were assessed before and after intervention; including arm, thigh, calf and breast circumferences, subscapular, triceps, biceps and suprailiac skinfold thicknesses, sum of skinfold thicknesses (SSF), body density (D) and estimated percent of body fat (EPF).

All anthropometric measurements were made on the right side of the body by the identical nutritionist in both the first and the follow-up assessments. Weight was measured without shoes while the participants had minimal cloths and recorded to the nearest 0.5 kg. Height was measured without shoes while the shoulders had a normal position.

The following circumferences were measured without any pressure to surface of the body by the light clothing using a tape measure, to the nearest 0.1 cm: 1) mid arm circumference at the level of the triceps skin-fold with the arm hanging relaxed at the subject’s side; 2) breast circumference at the level of the nipples; 3) thigh circumference directly below the gluteal fold; 4) calf circumference at the maximum level between the ankle malleoli and the knee; 5) waist circumference at the minimal level and 6) the hip circumference at the largest circumference. Skin-fold thicknesses were measured in a comfortable standing position while the body weight was distributed equally on both legs. A mean of three measurements was used. The following skinfold-thickness were measured with a Harpenden Skinfold Caliper (Baty international, England) to the nearest 0.5 mm: 1) skinfold thickness of biceps at the midpoint between the olecranon and acromion above the antecubital fossa in vertical state, 2) vertical skinfold thickness of triceps at the same level as the skinfold thickness of biceps, 3) oblique skinfold thickness of suprailiac in the midaxillary line on the iliac crest, and 4) the skinfold thickness of subscapular under the inferior end of the scapula.

Body mass index (BMI), waist to hip ratio (WHR), body density (D) and estimated percent of fat (EPF) were calculated using following formulas, respectively:

BMI = weight (kg)/stature (m)^2^

WHR= waist (cm)/hip (cm)

D = c -[m × log (sum of 4 skin fold thicknesses: triceps, biceps, subscapular and suprailiac)]^28^

EPF = 100 × [(4.95/D) -4.5]^28^

After 12 hours of fasting, blood samples were gathered and centrifuged at 4°C and 500 g for 10 minutes which separated the serum. Then serum was frozen at -80°C until it was analyzed for NO_x_.

Nitrites/nitrates were measured concurrently using the Griess reaction: first nitrates were reduced to nitrites by vanadium (III), and then total nitrites were measured.^29^

Additional covariate information regarding age, age of obesity initiation, education levels, income status, overweight and metabolic syndrome in family, family economic status, dieting history, number of dieting in past, dieting duration in past, weight loss in past diets, time of dieting and weight maintenance in the past diets was obtained by questionnaires. Because the chronic dieters would actually have less weight loss in hypocaloric diets, if they weren’t distributed among the groups, they could cause errors in the study. For this reason, dieting history, number of dieting in past, and dieting duration in past were considered as co-ariates. Subjects were considered in 3 education levels (under high school diploma, high school diploma and university graduates), 3 levels of income (no income (housekeeper), lower than 350 $ per month, and more than 350 $ per month), 3 levels of family income (lower than 350 $ per month, 350-700 $ per month, and more than 700 $ per month) and 3 levels of overweight and metabolic syndrome in family (anybody, first degree relatives, and second degree relatives). A participant was characterized as overweight if BMI was more than 25. Definition of metabolic syndrome was according to the Adult Treatment Panel III criteria.^30^

#### Statistical Analysis

Sample size for each group was estimated with regard to the previous studies conducted on obese women. With a 1-ß = 95% and 1-α = 95%, the maximum sample size was achieved from WC indicator. Finally samples for each group were evaluated to be 17 persons. All values are expressed as means ± SE at each time interval.

One-way ANOVA and *χ*^2^ tests were used to find out the significant differences of baseline values among diet groups. In appropriate variables, sub classes of variable were merged and then *χ*^2^ test was used. Nested M-ANOVA repeated measurements of multi-factor model was used with the following form which analyzed with Minitab Package V13 to recognize the effects of time and treatments and interaction between treatments on the different variables:

Variation of dependent variables = Persons + Time + Arg (Time) + Se (Time) + Arg × Se (Time) + Error.

“Person” represents the dependency of subjects within groups in different times. “Time” stand for the HDEL effect.

The end point measurements of each group was also compared with HDEL group using independent sample t test in SPSS for Windows version 13.0 (SPSS, Chicago, IL). Two-tailed p value less than 0.05 was considered significant.

## Results

The general characteristics of four groups have been shown in table 3. As it has outlined in table 3, the mean age of obesity initiation in all participants was 18.5 years. The education levels of the participants were 44% under high school diploma, 29% high school diploma and 27% university graduates. Most of the participants (82%) were housekeeper. Family income per month in 20% of the participants was less than 350 $, in 43% of them 350-700 $ and in 37% of them more than 700 $. Only 9% of the participants didn’t have overweight members in first and second degree relatives. Eighty two percent of participants had at least one overweight member in first degree relatives and 9% in second degree relatives. Forty four percent of subjects hadn’t metabolic syndrome history in first and second degree relatives while 45.6% had at least one member in their first degree relatives. The mean of dieting number and weight loss were 1 and 4.8 kg, respectively, but only 11% had maintained their weight loss. There were no differences in the general characteristics of 4 groups.

**Table 3:** The general characteristics of the four groups

	Treatments				
	HDEL	HDEL + Arg	HDEL + Se	HDEL + Arg + Se	P
N	17	17	17	17	-
Age (year) (mean ± SE)	36.6 ± 8.6	33.8 ± 9.1	36.7 ± 8.3	33.9 ± 8.5	NS
Height (cm) (mean ± SE)	158.7 ± 7	159.7 ± 6	157.1 ± 7	156.4 ± 6	NS
Age of obesity initiation (year) (mean ± SE)	17.5 ± 8.7	18.2 ±10.4	19.6 ± 8.7	18.5 ± 8.1	NS
Education (n, %):					NS
Under high school diploma	8 (47%)	10 (59%)	7 (41%)	5 (30%)	
High school diploma	4 (23%)	3 (18%)	6 (35%)	7 (41%)	
University graduates	5 (29%)	4 (23%)	4 (23%)	5 (29%)	
Income status (n, %):					NS
No income (Housekeeper)	12 (70%)	16 (94%)	15 (88%)	13 (76%)	
Less than 350 & per month	2 (12%)	0 (0%)	0 (0%)	3 (18%)	
More than 350 & per month	3 (18%)	1 (6%)	2 (12%)	1 (6%)	
Overweight in family (n, %):					NS
Anybody	1 (6%)	2 (12%)	2 (12%)	1 (6%)	
First degree relatives	13 (76%)	14 (82%)	15 (88%)	14 (82%)	
Second degree relatives	3 (18%)	1 (6%)	0 (0%)	2 (12%)	
Metabolic syndrome in family (n, %):					NS
Anybody	9 (53%)	8 (47%)	6 (35%)	7 (41%)	
First degree relatives	7 (41%)	6 (35%)	10 (59%)	8 (47%)	
Second degree relatives	1 (6%)	3 (18%)	1 (6%)	2 (12%)	
Family economic status (n, %):					NS
Less than 350 & per month	4 (23%)	5 (29%)	1 (6%)	4 (23%)	
350-700 & per month	7 (41%)	6 (35%)	6 (35%)	10 (59%)	
More than 700 & per month	6 (35%)	6 (35%)	10 (59%)	3 (18%)	
Dieting history (n, %):					NS
Yes	9 (53%)	10 (59%)	6 (35%)	7 (41%)	
No	8 (47%)	7 (41%)	11 (65%)	10 (59%)	
Number of dieting (n) (mean ± SE)	1.1 ± 1.6	0.6 ± 0.9	1.1 ± 1.4	0.8 ± 0.8	NS
Dieting duration (day) (mean ± SE)	253 ± 877	477 ± 1239	509±1871	248 ± 863	NS
Weight loss in dieting periods (kg) (mean ± SE)	5.3 ± 8.3	3.7 ± 6.6	4.4 ± 5	5.7 ± 6.6	NS
Time of dieting (n, %):					NS
Any time	8 (47%)	10 (59%)	6 (35%)	7 (41%)	
6 months to 1 year ago	3 (18%)	1 (6%)	6 (35%)	4 (23%)	
1 to 5 years ago	3 (18%)	4 (23%)	3 (18%)	4 (23%)	
More than 5 years ago	3 (18%)	2 (12%)	2 (12%)	2 (12%)	
Weight maintenance in past diets (n, %):					NS
No dieting	9 (53%)	9 (53%)	6 (35%)	7 (41%)	
Maintenance of reduction	0 (0%)	1 (6%)	1 (6%)	2 (12%)	
Some maintenance	0 (0%)	1 (6%)	2 (12%)	0 (0%)	
No maintenance	8 (47%)	6 (35%)	8 (47%)	8 (47%)	

HDEL: Hypocaloric Diet Enriched in Legumes; Arg: L-Arginine; Se: Selenium

Intake of food groups, calorie intake and calories expended in activity before run-in period are showed in table 4. The average intake of milk and fruit in all of the groups was low.

**Table 4:** Intake of food groups, calorie intake and calories expended in activity before run-in period.

	HDEL	HDEL + Arg	HDEL + Se	HDEL + Arg + Se	P
Basal milk intake (serving)	0.3 ± 0.6	0.4 ± 0.5	0.7 ± 0.4	0.6 ± 0.4	NS
Basal vegetable intake (serving)	2.3 ± 1.2	2.1 ± 1.3	1.9 ± 1	2.1 ± 1.5	NS
Basal fruit intake (serving)	1.5 ± 1.2	1.1 ± 1	1.4 ± 1	1.2 ± 0.8	NS
Basal meat intake (serving)	2.8 ± 1.6	3.2 ± 1.5	3.3 ± 1.8	3.8 ± 2.1	NS
Basal cereal intake (serving)	10.3 ± 3.8	10.6 ± 3.9	10.7 ± 4.1	11 ± 4	NS
Basal legumes intake (serving)	0.5 ± 0.5	0.3 ± 0.3	0.5 ± 0.4	0.4 ± 0.5	NS
Basal sugar intake (serving)	2.7 ± 1.1	3.2 ± 2	3.2 ± 1.9	3.4 ± 1.4	NS
Basal fat intake (serving)	13.3 ± 7.6	10.6 ± 4.1	15.3 ± 7.8	14.9 ± 12.3	NS
Basal activity calories (kcal)	337 ± 186	299 ± 123	440 ± 284	310 ± 233	NS
Basal calories intake (kcal)	2046 ± 725	1970 ± 615	2262 ± 792	2279 ± 901	NS

Values are means ± SE. HDEL: Hypocaloric Diet Enriched in Legumes; Arg: L-Arginine; Se: Selenium.

Groups had no differences in before run-in amounts of food intakes.

Anthropometric measures before intervenetion and 3 and 6 weeks after it were outlined in table 5. There were no significant differences among basal (before intervention) anthropometric measures in 4 groups (not shown in table 5).

**Table 5:** Evaluated parameters in different studied groups

	Treatment															
	HDEL			HDEL + Arg			HDEL + Se			HDEL + Arg + Se						
					
	T_1_ (mean ± SE)	T_2_ (mean ± SE)	T_3_ (mean ± SE)	T_1_ (mean ± SE)	T_2_ (mea n ± SE)	T_3_ (mean ± SE)	T_1_ (mean ± SE)	T_2_ (mean ± SE)	T_3_ (mean ± SE)	T_1_ (mean ± SE)	T_2_ (mean ± SE)	T_3_ (mean ± SE)	P_time_	P_Se_	P_Arg_	P_arg+Se_
Weigh (kg)	80.3 ± 1.7	79.1 ± 1.8	77.7 ± 1.9	80.4 ± 2.9	78.8 ± 2.9	78.1 ± 2.9	80.2 ± 2.3	78.6 ± 2.3	78 ± 2.4	80.3 ± 3.4	78.7 ± 3.3	77.8 ± 3.3	0.339	0.999	1	0.999
Waist (cm)	92.7 ± 1.7	89.8 ± 1.9	88.4 ± 1.8	92.2 ± 2.2	86.9 ± 2.3	84.8 ± 2.5	91.5 ± 1.8	87.9 ± 1.6	86.1 ± 1.5	91.9 ± 2.3	87.1 ± 2.5	85.9 ± 2.7	0.000	0.938	0.609	0.791
Hip (cm)	111.9 ± 2.6	110.2 ± 2.4	108.9 ± 2.5	112 ± 2	108.9 ± 2	108.1 ± 1.9	112.3 ± 2.4	109.4 ± 2.4	107.7 ± 2.4	111.5 ± 2.4	108.5 ± 2.1	107.1 ± 2.1	0.010	0.925	0.908	0.995
WHR	0.83 ± 0.01	0.82 ± 0.01	0.81 ± 0.01	0.82 ± 0.02	0.8 ± 0.02	0.78 ± 0.02	0.82 ± 0.02	0.81 ± 0.01	0.80 ± 0.01	0.82 ± 0.01	0.80 ± 0.02	0.81 ± 0.02	0.058	0.867	0.770	0.437
Arm (cm)	35.6 ± 0.9	-	34.2 ± 0.9	34.9 ± 1.1	-	32.8 ± 1.1	36.6 ± 0.9	-	34.7 ± 0.9	35.4 ± 0.9	-	34.4 ± 0.9	0.000	0.361	0.380	0.876
Thigh (cm)	69.1 ± 1.3	-	68.1 ± 2.2	69 ± 1.4	-	66.7 ± 1.4	69.8 ± 1.1	-	67 ± 1.2	70.2 ± 1.6	-	68.2 ± 1.4	0.000	0.866	0.997	0.672
Calf (cm)	41 ± 0.8	-	39.8 ± 0.7	41.1 ± 0.6	-	39.7 ± 0.5	41.8 ± 1	-	41.1 ± 0.7	41.6 ± 0.8	-	40.2 ± 0.7	0.000	0.268	0.862	0.916
Breast (cm)	108.1 ± 1.8	-	105.8 ± 1.8	109 ± 2.1	-	106.6 ± 2.3	108.9 ± 2	-	106.3 ± 2	109 ± 2.4	-	106.8 ± 2.3	0.000	0.992	0.974	0.994
Subscapular (mm)	25.9 ± 1.6	-	23.9 ± 1.5	25.6 ± 2.3	-	24.5 ± 1.9	25.5 ± 1.7	-	25.1 ± 1.7	25.8 ± 3.2	-	24 ± 2.6	0.000	0.995	0.999	0.967
Triceps (mm)	29.3 ± 2	-	25.5 ± 2.3	29.3 ± 1.5	-	25.6 ± 1.3	29.6 ± 1.7	-	25.3 ± 1.3	29 ± 2.3	-	24.8 ± 1.7	0.000	0.988	0.997	0.989
Biceps (mm)	18.1 ± 2	-	13.3 ± 1.6	18.8 ± 2	-	14.8 ± 1.1	18.3 ± 2	-	13.8 ± 1.1	18.6 ± 2.4	-	15.1 ± 1.3	0.000	0.993	0.755	0.998
Suprailia
c (mm)	20.3 ± 1.5	-	18.4 ± 1.5	20.9 ± 2.1	-	16.8 ± 1.7	20.8 ± 1.7	-	17.9 ± 1.7	20.3 ± 2.6	-	14 ± 1.4	0.000	0.719	0.283	0.863
SSF (mm)	93.6 ± 6	-	81 ± 5.8	94.8 ± 6.6	-	81.7 ± 4.3	94.1 ± 5.9	-	82.2 ± 4.9	93.8 ± 8.8	-	78 ± 5.9	0.000	0.995	0.986	0.962
D	1.017 ± 0.002	-	1.021 ± 0.002	1.0170 ± 0.002	-	1.0210 ± 0.001	1.016 ± 0.002	-	1.020 ± 0.002	1.018 ± 0.003	-	1.022 ± 0.002	0.000	1	0.838	0.860
EPF (%)	36.8 ± 0.95	-	34.7 ± 1	36.7 ± 0.8	-	34.8 ± 0.7	37.2 ± 1	-	35.4 ± 1	36.4 ± 1.4	-	34.3 ± 1.1	0.000	1	0.840	0.872
BMI (kg/m^2^)	32 ± 0.8	31.50 ± 0.8	30.9 ± 0.8	31.6 ± 1	31 ± 1	30.7 ± 1.1	32.4 ± 1.1	31.8 ± 1.1	31.5 ± 1.1	32.5 ± 1.4	31.8 ± 1.4	31.5 ± 1.4	0.429	0.802	0.995	0.990
NO_x_ (umol/l)	29.3 ± 8.2	37.2 ± 9.4	33 ± 7.4	29 ± 8	27.4 ± 6.5	29.6 ± 10.5	29.1 ± 16.3	21.6 ± 14.3	21.1 ± 11.2	29.4 ± 14.2	29.3 ± 12.9	23.5 ± 13.5	0.647	0.454	0.865	0.850

HDEL: Hypocaloric Diet Enriched in Legumes; Arg: L – arginine; Se: Selenium; WHR: Waist to Hip Ratio; SSF: Sum of Skin Fold Thicknesses; D: Body Density; EPF: Estimated Percent of body Fat; BMI: Body Mass Index; NOx: Nitrite/ Nitrate; T1: before intervention; T2: 3 Weeks After Intervention; T3: 6 Weeks After Intervention.

After 6 weeks that L-Arginine and selenium administration was added to HDEL; the following results were obtained (Table 5). 1) Statistically significant effect of HDEL in reduction of waist, hip, arm, thigh, calf and breast circumferences, triceps, biceps, subscapular and suprailiac skinfold thicknesses, WHR, SSF, D and EPF. 2) The effect of HDEL on reduction of weight and BMI. 3) Although the reduction of waist circumference in HDEL plus L-Arginine group and supra-iliac skin fold thickness in HDEL plus L-Arginine and selenium group was higher, but these differences were not significant in Nested M-ANOVA repeated measurements of multi-factor model. The difference between end of treatment values of suprailiac skinfold thickness in HDEL (20.3 ± 2.6) and HDEL + L-arginin + selenium (14 ± 1.4) groups was significant in independent sample t-test (p = 0.04). In this model L-Arginine and selenium added to HDEL significantly lowered the suprailiac skinfold thickness in comparison with HDEL. 4) There was no significant difference between treatments regarding variables.

## Discussion

Obesity is defined as a continual disparity between intake and expenditure of energy. It is interesting to find dietary supplementations which might return this balance. Different supplements are proposed to increase the energy expenditure and lower the body fat. In the present study, the purpose was to assess whether supplementation with L-Arginine and selenium, alone or together, could increase energy expenditure and modify body composition in central obese women.

In the current study, L-Arginine (5 gr/d), selenium (200 µg/d), or a combination of them that were added to hypocaloric diet enriched in legumes did not affect the anthropometric measures when it was used for 6 weeks. Only suprailiac skinfold thickness in HDEL + L-Arginine + selenium group reduced significantly compared with HDEL group in independent sample t-test; although its reduction in the stronger model (Nested M-ANOVA repeated measurements of multi-factor model) was not significant. Different results from two statistical methods necessitate using a more efficient method like bioelectrical impedance analysis (BIA) or computed tomography in measuring body fat.

Although L-Arginine and selenium were added at realistic levels (in terms of both typical dietary exposures), it could be argued that efficacy requires bolus dose rather than divided dose, still higher amounts or perhaps longer periods of consumption. It is very important to find out the lowest effective dose of L-Arginine. Previous studies showed that high doses of L-Arginine created atherosclerotic plaque by formation of peroxynitrit, oxidation of lipid and uncoupling of NO synthase.^31^,^32^

Our hypothesis was that L-Arginine lowers adipose tissue via L-Arginine-nitric oxide pathway. Also, L-Arginine is stimulus of growth hormone secretion. An increase in the peak of the growth hormone and the area under the curve was seen with increasing doses of L-Arginine.^33^ Using low doses of L-Arginine and selenium minimized growth hormone stimulating effect of oral L-Arginine and the detrimental effect of high levels of selenium.

The reduction of suprailiac skinfold thickness in HDEL + L-Arginine + selenium group proved this hypothesis. Although low dose of oral L-Arginine (5 gr/d) did not increase fasting NO_x_ concentrations, probably, it has enhanced transiently the concentrations of NO_x_ in serum after consumption and selenium has increased the availability of NO. In this study, the participants were enrolled from a population whose mean serum selenium concentrations were lower than 80 µg/dl. In the previous studies selenium deficiency enhanced the expression of iNOS.^23^,^24^ Hence, selenium supplementation could significantly increase availability of NO and decrease the expression of iNOS.

L-Arginine plus selenium reduced the suprailiac skinfold thickness which was representative of abdominal obesity. In consistence with this study in Khedara et al study lower concentrations of NO had a greater effect on increasing abdominal adipose tissues than the subcutaneous adipose tissues in rats.^34^

In animals high dose of L-Arginine (1.25% L-Arginine in drinking water) reduced fat mass and increased serum levels of NO metabolites in ZDF rats,^8^ reduced white fat gain and increased serum levels of NO metabolites in diet-induced obese rats,^9^ reduced retroperitoneal fat mass in Sprague Dawley rats^35^ and reduced body fat mass in growing-finishing pigs.^10^ Khedara et al showed that L-N -nitroarginine (L-NNA), dietary NOS inhibitor, increased body fat in wistar rats due to reduction of fatty acid oxidation.^34^ Fu et al hypothesized that L-Arginine could increase oxidation of macronutrients and the loss of adipose tissue because of increasing NO production within physiologic ranges. They showed that oral L-Arginine supplementation promoted expression of the following main genes involved in oxidation of glucose and fatty acid in adipose tissue: AMP-activated protein kinase, NO synthase-1, peroxisome proliferators, activated receptor coactivator-1α and heme oxygenase-3.^8^ However, Tsuchiya et al showed that chronic block of NOS decreased fat mass in standard diet mice and high fat-induced obese mice. NG-nitro-L-Arginine methyl ester (L-NAME) treatment up-regulated the uncoupling protein-1 in the brown adipose tissue of high fat diet (HFD) mice. Moreover, L-NAME also increased peroxisome proliferators-uncoupling protein-3 mRNA levels in skeletal muscles of HFD-fed mice. In L-NAME-treated mice increased urinary excretion of norepi-nephrine after HFD feeding was augmented.^36^ Fat mass losing effect of both NO substrate (L-Arginine) in Jobgen et al^9^ study and NOS inhibitor (L-NAME) in Tsuchiya et al^36^ study, in diet-induced obese rats, implicate a true resulting. L-arginine and L-NAME effects on fat mass may be mediate by another ingredient other than NO.

In a recent human study by Luccoti et al 8.3 g/d oral L-Arginine supplement in 3 weeks, significantly reduced WC and fat mass and increased NO_x_ incremental area (umol/1-6 minutes) in insulin resistant, obese type 2 diabetic patients but did not have significant effect on weight.^11^ In a research by Martina et al L-Arginine intravenous administration (1200 mg Zentrum, one vial a day) plus N-acetylcysteine (600 mg Acetilcisteina, one tablet twice a day) in 6 months increased NO_x_ significantly in patients with hypertension and type 2 diabetes.^37^ Piatti et al performed a study on the patients with type 2 diabetes and showed L-Arginine (9 g/d) in 4 weeks had no effect on weight.^38^ In another study on patients who had a coronary bypass surgery, L-Arginine (6.4 g/d) did not affect weight, fat mass, fat free mass, WC, and NO_x_.^39^ The beneficial effect of Lucotti’s study in reducing fat mass and WC could be related to higher dose of L-Arginine, and type of their participants, who were diabetic.

In most of the human studies, L-Arginine supplementation had no effect on serum NO_x_ levels.^39^–^43^ Studies in which L-Arginine increased NO levels had one of the following conditions: 1) intravenous administration of L-Arginine^37^ ; 2) oral supplementation in peripheral atherosclerotic patients^44^ ; and 3) measure of NO_x_ incremental area in 1 to 10 minute after oral supplementation.^11^

This is the first time that the simultaneous effects of hypocaloric diet, L-Arginine and selenium on anthropometric indices are studied. The advantage of the present study was its statistical method. Although, each group had 17 cases, with Nested M-ANOVA repeated measurements of multi-factor model, simultaneous analysis was used on 68 cases. The concurrency of all analyses in this model minimized the probability of false positive results due to multiple comparisons. The net effects of interventions were separated by omitting the effect of intra-individual variability in participants and random changes during the study by entering “Person” and “Error” in the statistical model. There were several limitations. First, the estimation of body fat with advanced procedures was not possible. Second, serum levels of L-Arginine and selenium were not measured. Third, using a basic hypocaloric diet impacted net effect of treatments. Fourth, we couldn’t provide food to participants.

## Conclusions

The purpose of this research was to study the effects of dietary supplementation on anthropometric measures in central obese women. Any effect of L-Arginine and selenium, alone or together, was not observed. Only suprailiac skinfold thickness in HDEL + L-Arginine + selenium group was reduced significantly compared to HDEL group in independent sample t-test. Further studies, with different doses of supplements in obese diabetic and healthy people, are necessary to make true conclusions.
